# Acute telogen effluvium triad after resolution^☆^^☆☆^

**DOI:** 10.1016/j.abd.2020.10.008

**Published:** 2021-07-14

**Authors:** Leticia Arsie Contin, Vanessa Barreto Rocha

**Affiliations:** a Dermatology Clinic, Hospital do Servidor Público Municipal, São Paulo, SP, Brazil; b Hospital das Clínicas, Belo Horizonte, Minas Gerais, MG, Brazil

**Keywords:** Alopecia, Dermatology, Diagnosis, Hair

## Abstract

Five cases of telogen effluvium undergoing resolution are shown, with the presence of frontal, bitemporal, and occipital hair regrowth. Diagnosing acute telogen effluvium after the end of the active phase can be challenging, especially when the pull test is negative. The differential diagnosis includes alopecia areata and traction alopecia. Clinical signs of hair regrowth after telogen effluvium can help in the diagnosis. The frontal and temporal areas have more telogen hairs and are more affected. On the occipital area, hairs seem to have the same behavior. The acute telogen effluvium triad during resolution is proposed: frontal fringe, temporal recess and occipital fringe.

## Case Reports

Case 1: Female patient, 35 years old, healthy. She had hair loss three months after pregnancy, which lasted three months, followed by spontaneous hair regrowth. The picture was taken 15 months after delivery.

Case 2: Female patient, 51 years old who, three months before the appointment, had severe hair loss after losing weight. The picture shows the hair status three months after the hair loss stopped.

Case 3: Female patient, 45 years old. After losing weight on a restrictive diet, she had significant hair loss and was seen in consultation three months after the loss stopped.

Case 4: Female patient, 34 years old. After a serious car accident, she was hospitalized for an extradural hematoma and amputation of her arm. After three months, she had severe hair loss. The picture shows the patient four months after stabilization of her condition.

Case 5: Female patient, 17 years old, diagnosed with neurofibromatosis, hospitalized due to a severe intestinal infection. She had severe hair loss three months after hospital discharge, which resolved spontaneously. The picture was taken at the consultation three months after hair loss stopped.

**Figure 1:**
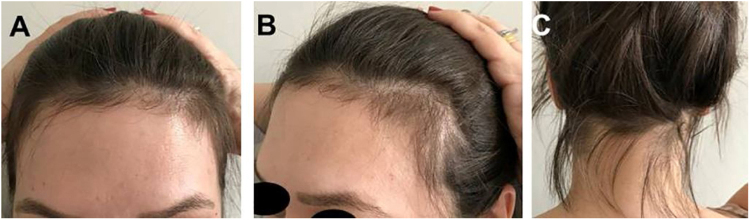
Case 1: (A), Frontal fringe. (B), hair rarefaction on the temporal region and (C), occipital fringe of hair regrowth after effluvium in the post-partum period.

**Figure 2:**
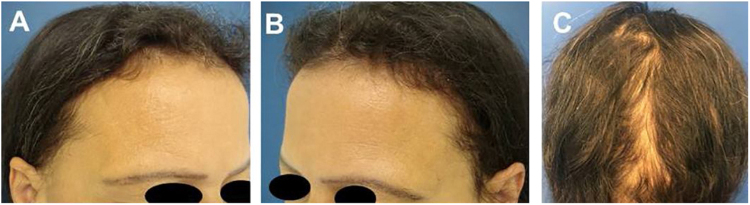
Case 2: (A), Frontal fringe. (B), hair rarefaction on the temporal region and (C), occipital fringe hair regrowth after effluvium post-weight loss.

**Figure 3:**
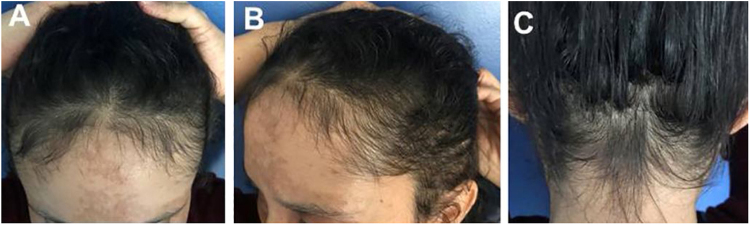
Case 3: (A), Frontal fringe. (B), Hair rarefaction on the temporal region and (C), occipital hair regrowth fringe after effluvium post-weight loss.

**Figure 4:**
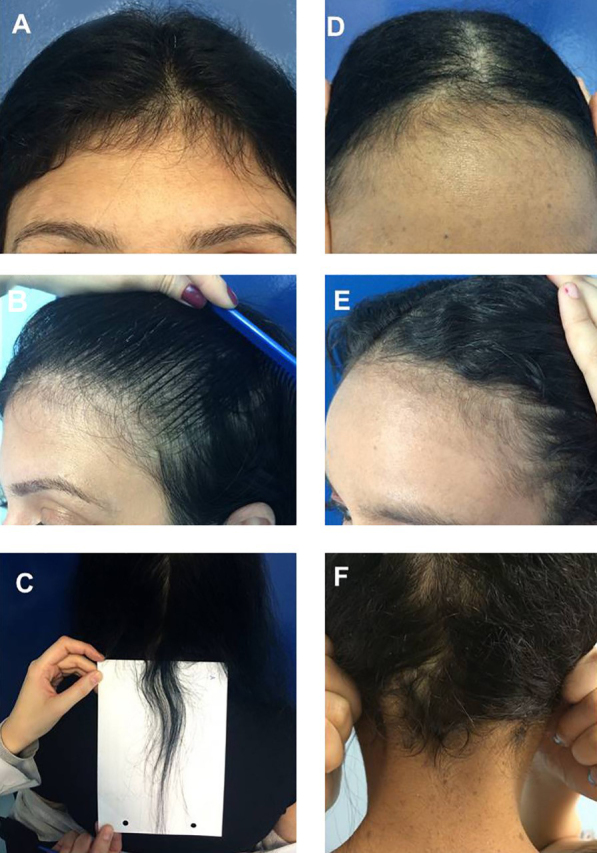
Case 4: (A), Frontal fringe. (B), hair rarefaction on the temporal region and (C), occipital hair regrowth fringe after a serious car accident; Case 5: (D), Frontal fringe, (E), temporal rarefaction and (F), occipital hair regrowth fringe post-effluvium after severe intestinal infection.

Figs. 1 to 4 show these five cases of telogen effluvium, with a significant amount of hair regrowth hairs in the frontal, bitemporal and occipital regions.

**Figure 5:**
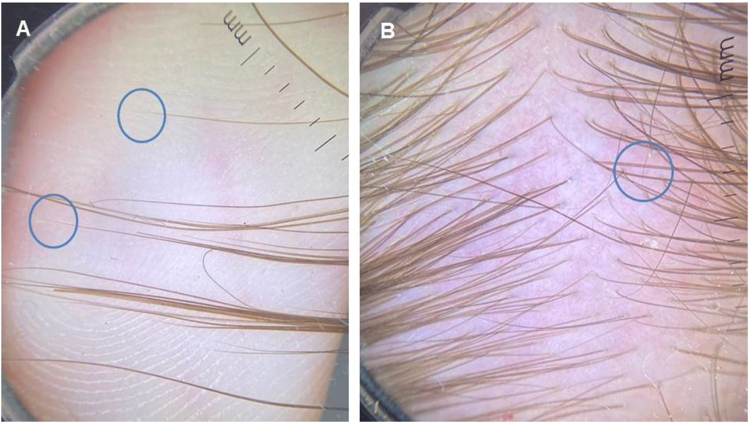
Analysis of hair ends: (A), the tapered ends correspond to short regrowing hairs. (B), Trichoscopy of the occipital area shows several short hairs with tapered ends.

Fig. 5 shows an analysis of the hair ends: the tapered ends correspond to short regrowing hairs. Trichoscopy of the occipital area revealed several short hairs whose ends show these characteristics.

## Discussion

Diagnosing acute telogen effluvium (TE) after its active phase is over can be a challenge in many cases, especially when the pull test is already negative. The question is whether there is actually any hair loss, if there was a recent effluvium or if the patient (usually female) has an increased perception of this situation.

The diagnosis of TE is essentially clinical, as laboratory tests, trichoscopy or histopathological alterations usually do not confirm the diagnosis.^1^ A trichogram may be helpful when more than 20% of telogen hairs are present in it.^2^, ^3^ The main differential diagnosis includes alopecia areata, as substantial hair loss can be diffuse or occur in localized areas.^4^, ^5^ In active diffuse alopecia areata the mild pull test is usually positive for anagens, the trichogram may show dystrophic anagen hairs, trichoscopy shows black and yellow dots, exclamation hairs, and there may be short regrowing hairs. The histopathological analysis may show lymphocytic peribulbitis in the acute phase.^5^

Traction alopecia may also be included in the clinical differential diagnosis, with the fringe sign present on the side undergoing traction.^6^ The trichogram is normal, trichoscopy shows vellus hairs, peripilar cylinders, black dots, and broken hairs, and the histopathological analysis shows preserved sebaceous glands, increased number of telogen and catagen hairs, increased vellus and decreased terminal hairs, in addition to trichomalacia and pigment clumps.^7^

The differential diagnosis can also be made with frontal fibrosing alopecia, ruled out by the presence of numerous vellus hairs in the region of hair implantation, and with female androgenetic alopecia, not confirmed by the absence of hair miniaturization in the presented cases.^8^, ^9^

Knowledge of the clinical signs of hair regrowth after telogen effluvium can help in this differential diagnosis. As the frontal and temporal regions of the scalp have a greater number of telogen hairs, these regions seem to be more affected by this process.^10^ Moreover, the hairs on the occipital region, although there is no description of the fact in the literature to date, also seem to show a predominance of telogen hairs. This set of signs, consisting of temporal rarefaction, frontal and occipital fringe, is called the acute telogen effluvium triad after resolution (Figs. 1 to 4). It can help to clinically differentiate which patients are actually undergoing TE or have recently had it. Trichoscopy shows several hairs undergoing regrowth (Fig. 5).

The knowledge of this triad can help in the diagnosis of TE and the careful management of patients, with the proposal of a conservative treatment, without the need for more invasive complementary exams, such as a scalp biopsy, for instance.

## Financial support

None declared.

## Authors' contributions

Leticia Arsie Contin: Approval of the final version of the manuscript; design and planning of the study; drafting and editing of the manuscript; collection, analysis, and interpretation of data; effective participation in research orientation; intellectual participation in propaedeutic and/or therapeutic conduct of the studied cases; critical review of the manuscript.

Vanessa Barreto Rocha: Approval of the final version of the manuscript; drafting and editing of the manuscript; analysis and interpretation of data; critical review of the literature; critical review of the manuscript.

## Conflicts of interest

None declared.

## References

[1] Mubki T., Rudnicka L., Olszewska M., Shapiro J. (2014). Evaluation and diagnosis of the hair loss patient: part I. History and clinical examination. J Am Acad Dermatol..

[2] Pereira J.M. (1993). The trichogram Part I. Significance and method of performing. An Bras Dermatol..

[3] Pereira J.M. (1993). The trichogram: Part II. Results and interpretation. An Bras Dermatol..

[4] Werner B., Mulinari-Brenner F. (2012). Clinical and histological challenge in the differential diagnosis of diffuse alopecia: female androgenetic alopecia, telogen effluvium and alopecia areata - part I. An Bras Dermatol..

[5] Werner B., Mulinari-Brenner F. (2012). Clinical and histological challenge in the differential diagnosis of diffuse alopecia: female androgenetic alopecia, telogen effluvium and alopecia areata--part II. An Bras Dermatol..

[6] Samrao A., Price V.H., Zedek D., Mirmirani P. (2011). The "Fringe Sign" - A useful clinical finding in traction alopecia of the marginal hair line. Dermatol Online J..

[7] Billero V., Miteva M. (2018). Traction alopecia: the root of the problem. Clin Cosmet Investig Dermatol..

[8] Lacarrubba F., Micali G., Tosti A. (2013). Absence of vellus hair in the hairline: a videodermatoscopic feature of frontal fibrosing alopecia. Br J Dermatol..

[9] Ramos L.D., Santili M.C., Bezerra F.C., Ruiz M.eF., Petri V., Patriarca M.T. (2012). Dermoscopic findings in female androgenetic alopecia. An Bras Dermatol..

[10] Pecoraro V., Astore I., Barman J., Ignacioaraujo C. (1964). The normal trichogram in the child before the age of puberty. J Invest Dermatol..

